# Novel Formulations of C-Peptide with Long-Acting Therapeutic Potential for Treatment of Diabetic Complications

**DOI:** 10.3390/pharmaceutics11010027

**Published:** 2019-01-11

**Authors:** Natalia Zashikhina, Vladimir Sharoyko, Mariia Antipchik, Irina Tarasenko, Yurii Anufrikov, Antonina Lavrentieva, Tatiana Tennikova, Evgenia Korzhikova-Vlakh

**Affiliations:** 1 Institute of Macromolecular Compounds, Russian Academy of Sciences, Saint-Petersburg 199004, Russia; nzashihina@bk.ru (N.Z.); volokitinamariya@yandex.ru (M.A.); itarasenko@list.ru (I.T.); 2 Institute of Chemistry, Saint-Petersburg State University, Saint-Petersburg 198584, Russia; sharoyko@gmail.com (V.S.); anufrikov_yuri@mail.ru (Y.A.); tennikova@mail.ru (T.T.); 3 Institute of Technical Chemistry, Leibniz University, Hannover 30167, Germany; lavrentieva@iftc.uni-hannover.de

**Keywords:** polypeptides, amphiphilic random copolymers, nanoparticles, C-peptide, encapsulation, diabetes

## Abstract

The development and application of novel nanospheres based on cationic and anionic random amphiphilic polypeptides with prolonged stability were proposed. The random copolymers, e.g., poly(l-lysine-*co*-d-phenylalanine) (P(Lys-*co*-dPhe)) and poly(l-glutamic acid-*co*-d-phenylalanine) (P(Glu-*co*-dPhe)), with different amount of hydrophilic and hydrophobic monomers were synthesized. The polypeptides obtained were able to self-assemble into nanospheres. Such characteristics as size, PDI and ζ-potential of the nanospheres were determined, as well as their dependence on pH was also studied. Additionally, the investigation of their biodegradability and cytotoxicity was performed. The prolonged stability of nanospheres was achieved via introduction of d-amino acids into the polypeptide structure. The cytotoxicity of nanospheres obtained was tested using HEK-293 cells. It was proved that no cytotoxicity up to the concentration of 500 µg/mL was observed. C-peptide delivery systems were realized in two ways: (1) peptide immobilization on the surface of P(Glu-*co*-dPhe) nanospheres; and (2) peptide encapsulation into P(Lys-*co*-dPhe) systems. The immobilization capacity and the dependence of C-peptide encapsulation efficiency, as well as maximal loading capacity, on initial drug concentration was studied. The kinetic of drug release was studied at model physiological conditions. Novel formulations of a long-acting C-peptide exhibited their effect ex vivo by increasing activity of erythrocyte Na^+^/K^+^-adenosine triphosphatase.

## 1. Introduction

Diabetes is one of the most common socially significant and chronic diseases worldwide. Diabetes and its complications are a major cause of morbidity and mortality. In this regard, the development of new approaches to prevent and treat the diabetic complications is one of the urgent problems of modern pharmacology and biomedical chemistry. In recent years, it has been reported that C-peptide (connecting peptide) can be used for the treatment of diabetic complications and therefore this biologically active peptide has attracted worldwide attention [1,2].

C-peptide, 31-amino acid peptide (EAEDLQVGQVELGGGPGAGSLQPLALEGSLQ), is a byproduct of proinsulin proteolysis in which it connects A and B chains of insulin [2]. The C-peptide provides the correct spatial assembly and packing of proinsulin molecule into the endoplasmic reticulum of insulin-secreting beta cells of pancreatic Langerhans islets. The newly synthesized insulin and C-peptide are stored in equimolar amounts in secretory granules and are released into the systemic blood stream as the blood glucose concentration increases. The role of insulin in the control of carbohydrate-fat metabolism is well known, whereas the role of C-peptide in the regulation of microvascular blood flow has been recognized relatively recently [3,4]. The C-peptide also has an anti-inflammatory effect, it is involved in the repair of lesions of smooth muscle cells; on the models of animals with type 1 diabetes it was shown that the administration of C-peptide normalized the function of nervous and excretory systems [5,6,7]. A decrease in the concentration of C-peptide or its complete absence is associated with the development and progression of severe complications of diabetes mellitus (strokes, heart attacks, blindness, limb amputation, renal failure) [8]. C-peptide replacement together with the classic insulin therapy may prevent, retard or ameliorate diabetic complications in patients with type-1 diabetes [9].

First results on the C-peptide efficacy were demonstrated in the end of 1990s [10]. It was established that twice a day C-peptide injections to rats with type 1 diabetes during 1.5–3 months at dose 400 μg/kg prevented to a large extent the development of renal, sciatic nerve and aortic vascular disorders and partially restored nerve conduction in muscles. To explore neuroprotective activity of C-peptide on type 1 diabetic neuropathy in rats Zhang et al. tested a range of day doses equal to 10, 100, 500 and 1000 μg/kg during 2 months administration [11]. It was established that 100 μg/kg was enough to prevented the nerve conduction defect.

However, as many other peptide drugs, C-peptide has low stability in vivo and demonstrates also rapid inactivation upon storage. It is known that the half-life of C-peptide in plasma is about 30 min in healthy humans and about 40 min in those with diabetes [12]. Therefore, to maintain plasma concentrations for as long as possible in clinical trials it has been necessary to administer the C-peptide for several times daily. Recently, the positive example on preparation of a long-acting form of C-peptide has been reported by Wahren et al. [13,14]. In the developing medicinal formulation, the authors used well-known approach to increase the resistance of peptide drug via its conjugation with PEG. The treatment of mice with a type 1 diabetic model twice a week for 20 weeks with PEG-C-peptide conjugate at a dose of 0.1–1.3 mg/kg was more efficient comparatively to the control treatment of animals with a native C-peptide. It is important that experiment with native C-peptide required more frequent treatment (twice a day) during the same period and at the same dose [14]. Once-weekly subcutaneous administration of PEG-C-peptide at doses of 0.8 mg and 2.4 mg during 12 months (tested for 250 patients) resulted in marked improvement of VPT (vibration perception threshold) in comparison to placebo injections [15].

Besides the preparation of PEG-peptide conjugates to improve the peptide circulation half-life in vivo and, consequently, to diminish the dose necessary for efficient administration, the application of polymer particles for peptide drug delivery can be also matter of choice [16,17,18]. Despite the fact that the works devoted to the development of efficient delivery systems of C-peptide were not discovered, several examples on preparation of encapsulated forms of insulin can be found in the current literature [19,20,21,22,23]. The polymer particles of different nature were applied to encapsulate insulin. Microspheres based on Eudragit S-100 copolymer [19], self-assembled chitosan-pectin nano- and microparticles [20], polymersomes based on dextran-*b*-poly(lactic acid-*co*-glycolic acid) [22], microspheres prepared from poly(glycolic acid) [24] were described as successful systems for the development of insulin delivery systems. The efficiency of insulin encapsulation was varied from 32 to 90%. The release explorations in model physiological conditions (PBS, pH 7.4) showed the fast release profiles. For example, insulin release from Eudragit S-100 particles was finished in 8 h and reached almost 100% [19]. In turn, insulin release from dextran-*b*-poly(lactic acid-*co*-glycolic acid) polymersomes took about 9 h to reach the level of 70–85% depending on the polymer composition [22].

The development of novel prospective C-peptide delivery systems undoubtedly represents an actual research goal, firstly, because of high great potential of C-peptide in the treatment of diabetes complications and, secondly, because of absence of commercially available long-acting C-peptide formulations that allow a decrease of administration frequency. Thus, the aim of this study was the creation of the new C-peptide formulations with extended life-time and tissue bioavailability. For this purpose, two different approaches have been suggested and realized: (1) covalent modification of polymer nanosphere’s surface with C-peptide; and (2) encapsulation of C-peptide into the developed polymer systems. In our case, synthetic random polypeptides were chosen as the polymers. The interest to synthetic polypeptides is induced by their biocompatibility, biodegradability and the possibility to vary the functional groups in a wide range [25]. The diversity of amino acids allows for the tuning of polymer properties such as hydrophilic/hydrophobic or charge/neutral ones. Since C-peptide is negatively charged molecule we selected P(l-lysine-*co*-d-phenylalanine) (P(Lys-*co*-dPhe) for peptide encapsulation. P(l-glutamic acid-*co*-d-phenylalanine) (P(Glu-*co*-dPhe) nanospheres were chosen for covalent modification with target peptide. An introduction of d-phenylalnine instead of its coding l-isomer into the polypeptide chain should improve the stability of nanospheres regarding to their biodegradation.

It is also known that biological activity of C-peptide belongs to its C-terminal fragment responsible for binding to the cell membrane [26]. Taking this into account, the short C-terminal EGSLQ pentapeptide was additionally synthesized and biological effect of its long-active forms was compared to that observed for the whole length peptide molecule. It is known that C-peptide improvement of human vascular blood flow in type 1 diabetes is mediated through a mechanism involving erythrocyte Na^+^/K^+^-ATPase activation [27]. In our work, to test the biological activity of different C-peptide forms the method of microcalorimetric titration was used to monitor the activation of Na^+^/K^+^-adenosine triphosphatase (Na^+^/K^+^-ATPase) [28].

## 2. Materials and Methods

### 2.1. Materials

d-phenylalanine (d-Phe), γ-benzyl-l-glutamic acid (Glu(OBzl), ε-carboxybenzyl-l-lysine (Lys(Z)), triphosgene, α-pinene, *n*-hexylamine (HEXA), trifluoromethanesulfonic acid (TFMSA), trifluoroacetic acid (TFA), *N*-hydroxysuccinimide (NHS), 1-ethyl-3-(3-dimethylaminopropyl) carbodiimide (EDC), PAM-resin (0.75 µmol/g) for solid phase peptide synthesis were delivered from Sigma-Aldrich (Darmstadt, Germany) and used as received. Recombinant insulin C-peptide was purchased from Bachem (Bubendorf, Switzerland). Ouabain specific inhibitor of Na^+^/K^+^-ATPase was delivered from Sigma-Aldrich (Darmstadt, Germany). Amino acid BOC-derivatives were the products of Iris Biotech (Marktredwitz, Germany). 1,4-dioxane, *n*-hexane, *N*,*N*-dimethylformamide (DMF), dimethyl sulfoxide (DMSO), tetrahydrofuran (THF), ethyl acetate, methanol, dichloromethane and other solvents were purchased from Vecton Ltd. (St. Petersburg, Russia) and distilled before use. All salts used for buffers preparation were also purchased from Vecton Ltd. and were of ACS reagent grade. The buffer solutions were prepared by dissolving salts in distilled water and additionally purified by filtration through a 0.45-μm membrane microfilter Milex, Millipore Merck (Darmstadt, Germany). Amicon membrane tubes used for ultrafiltration (MWCO 30,000) were the products of Merck (Darmstadt, Germany). The Spectra/Pore^®^ (MWCO: 1000) dialysis bags were purchased from Spectra (Rancho Dominguez, CA, USA).

### 2.2. Methods

#### 2.2.1. Synthesis and Polymer Characterization

The synthesis of random polypeptides was carried out by ring-opening polymerization (ROP) of α-amino acid *N*-carboxyanhydrides (NCA). NCA monomers of Lys(Z) and Glu(OBzl) (Figure S1 of Supplementary Materials) were prepared as described elsewhere [29]. Dioxane was used as a solvent for Lys(Z) and THF as a solvent for Glu(OBzl) and d-Phe synthesis. Acquired NCAs were purified by recrystallization from ethyl acetate/*n*-hexane. Yields: Lys(Z) NCA—57%, Glu(OBzl) NCA—83%, d-Phe NCA—42%.

The structure and purity of NCAs obtained were proved by ^1^H NMR at 25 °C in СDCl_3_. The spectra were recorded at 298 K using a Bruker 400 MHz Avance instrument (Karlsruhe, Germany). Lys(Z) NCA: δ 7.43–7.28 (m, 5H), 6.97 (s, 1H), 5.12 (s, 2H), 4.97 (s, 1H), 4.32–4.23 (t, *J* = 5.2, 1H) (s, 1H), 3.29–3.14 (m, 2H), 2.03–1.90 (m, 1H), 1.90–1.75 (m, 1H), 1.73–1.28 (m, 4H); Glu(OBzl) NCA: 2.05–2.39 (m, 2H), 2.63 (t, 2H), 4.39 (t, 1H), 5.17 (s, 2H), 6.40 (br. s., 1H), 7.39 (m, 5H); d-Phe NCA: 2.94–3.35 (m, 2H), 4.55 (m, 1H), 6.12 (s, 1H), 7.19–7.41 (m, 5H).

P(Lys(Z)-*co*-dPhe) and P(Glu(OBzl)-*co*-dPhe) were synthesized by ROP of corresponding NCAs using hexylamine as initiator. The molar ratio of Glu(OBzl)/Lys(Z) NCA to d-Phe NCA was 1/1, 4/1 and 8/1. The total NCAs/initiator molar ratio was 100/1 for Lys-containing polymers and 50/1 for Glu-based polymers. The synthesis was carried out in 1,4-dioxane or THF using 4 wt.% solution of NCA at 25 °C for 48 h. The product was precipitated with an excess of diethyl ether, the precipitate was washed three times with diethyl ether and then dried. Molecular weight characteristics of P(Lys(Z)-*co*-dPhe) and P(Glu(OBzl)-*co*-dPhe) were determined by size-exclusion chromatography (SEC). SEC was performed with the use of Shimadzu LC-20 Prominence system supplied with refractometric RID 10-A detector (Kyoto, Japan) using 7.8 mm × 300 mm Styragel Column, HMW6E, 15–20 µm bead size (Waters, Milford, MS, USA). The analysis was carried out at 60 °C using 0.1 M LiBr in DMF as eluent. The mobile phase flow rate was 0.3 mL/min. Molecular weights (*M_w_* and *M_n_*) and dispersity (*Đ*) for all synthesized polymers were calculated using GPC LC Solutions software (Shimadzu, Kyoto, Japan) and calibration curve built for poly(methyl methacrylate) standards with *M_w_* range from 17,000 to 250,000 and *Đ* ≤ 1.14.

The deprotection of *ε*-NH_2_-groups of P(Lys(Z)-*co*-dPhe) and γ-carboxylic groups of P(Glu(OBzl)-*co*-dPhe) was carried out using TFMSA/TFA mixture in a ratio 1/10 at 25 °C for 3 h. After removing protective groups, the products were precipitated with an excess of diethyl ether, the precipitates were washed three times with diethyl ether, dried and then dispersed in DMF. The suspensions of amphiphilic random copolymers were transferred into dialysis membrane bag MWCO 1000 and dialyzed against water for 48 h.

The contribution of hydrophobic part was determined using HPLC amino acid analysis after total hydrolysis of the samples. The hydrolysis of 3 mg of a sample was carried out in 6 mL of 6 M HCl with 0.0001% phenol in vacuum-sealed ampoule for four days. The solvent was evaporated several times with water to eliminate HCl and to reach finally the neutral pH value. The hydrolysates were analyzed using LCMS-8030 Shimadzu system with triple quadruple mass-spectrometry detection (LC-MS) (all from Shimadzu, Japan) equipped with 2 × 150 mm Luna C18 column packed with 5 μm particles. The isocratic elution mode was applied and 0.1% acetonitrile/НСООН in a ratio 5/95 wt.% was used as eluent. The mobile phase flow rate was equal to 0.3 mL/min.

#### 2.2.2. Preparation and Characterization of Nanospheres

Polymer nanospheres were prepared by phase inversion during dialysis. For this, polymer sample dissolved in DMSO was placed into dialysis bag with MWCO 1000 which then was deposited into the mixture of DMSO/water = 1/1 (*v*/*v*). After 2 h the solution was replaced with mixture DMSO/water = 1/3 (*v*/*v*) (2 h) and then with water (8 h). Self-assembled nanospheres were freeze-dried with the use of VaCo 5-II lyophilic system (Zirbus, Germany) and stored at 4 °C. Before use, the necessary amount of polymer nanospheres was placed in the medium of choice and redispersed by short-time ultrasonication (30–60 s) by means of ultrasonic probe UP 50H Hielscher Ultrasonics (Teltow, Germany). Average hydrodynamic diameter and polydispersity index (PDI) of polymer nanospheres were measured using dynamic light scattering (DLS) method and Zetasizer Nano-ZS (Malvern Instrument Ltd., Malvern, UK) equipped with a He–Ne laser beam at 633 nm and a detection angle of 173° using the samples with concentration of nanoparticles in the range 0.1–0.5 mg/mL. ζ-Potential was measured for 0.1 mg/mL colloids in distilled water containing 10^−3^ М NaCl and adjusted with 0.1 M HCl/NaOH to pH 2–12.

The morphological peculiarities were investigated using transmission electron microscopy (TEM) with a Jeol JEM-2100 (Tokyo, Japan) microscope operated at an acceleration voltage of 160 kV. Before analysis, a few drops of a sample were placed onto a copper grid covered with carbon for 30 s. The dried grid was stained negatively with 2% (*w*/*v*) uranyl acetate solution for 30–60 s and used for measurements after 24 h.

#### 2.2.3. Synthesis of C-Terminal Fragment of C-Peptide

C-terminal pentapeptide (27–31 fragment of C-peptide) H-Glu-Gly-Ser-Leu-Gln-OH (EGSLQ) was synthesized via solid phase peptide technique based on application of BOC/OBzl amino acid derivatives. The peptide cleavage from a resin was carried out using TFMSA/TFA system containing 1,2-ethanedithiol and thioanisole as scavengers. The program of synthesis was the same as published earlier [30]. The peptide was purified by size-exclusion chromatography with the use of Sephadex G-75 column (25 × 500 mm) and 6% acetic acid aqueous solution. The RP-HPLC analysis of synthesized product was performed at flow rate 1.0 mL/min using 4.6 × 150 mm Grace Smart C18 column packed with 5 µm particles and 0.1% H_3_PO_4_/H_2_O as buffer A and 0.1% H_3_PO_4_/AcN as buffer B. A linear gradient of 1 to 30% B in 25 min was applied. MS analysis was performed using MALDI-spectrometer Axima–Resonance Shimadzu (Kiyoto, Japan). The registered molecular ion was found to be [М + Н]^+^ = 533.54, which is consistent with the expected mass of the peptide (532.55 g/mol).

#### 2.2.4. In Vitro Biodegradation Study

Before the biodegradation studies all solutions were filtered through the sterile syringe PES membrane filters with pores of 0.45 μm (Membrane Solutions, Kent, WA, USA). The degradation process of P(Lys-*co*-dPhe) nanospheres was studied in model medium consisting of 0.01 M PBS, pH 7.4, and papain. Briefly, 500 μg of enzyme (papain, ~30 U/mg) were introduced into 1.0 mL of suspension containing 1.0 mg of nanospheres. The experiment was carried out during 35 days. Every several days, the probe of 20 μL of supernatant was sampled for HPLC monitoring of free amino acids (Lys and d-Phe). The commercially available ultra-short monolithic column, namely, CIM-SO3 disk of 3 mm × 12 mm i.d. (BIA Separations, Ajdovscina, Slovenia) was applied as a stationary phase. HPLC experiments were performed with the use of Shimadzu Liquid Chromatographic System LC-20AD (Canby, OR, USA). The data was acquired and processed with LS Solution software (Shimadzu, Kyoto, Japan). UV detection was performed at 210 nm. 0.02 M aqueous Na-acetic buffer, pH 3.7 (eluent A), 0.02 M Na-phosphate buffer, pH 7.0 (eluent B) and 0.0125 M Na-borate buffer, pH 10.0 (eluent C) were used as the components of a mobile phase. The separation was carried out at flow rate 0.5 mL/min following the gradient program: 0–0.5 min—eluent A, 0.5–7 min—eluent B, 7–10 min—eluent C.

#### 2.2.5. Particle Surface Modification with Peptides

The modification of the surface of P(Glu-*co*-dPhe) nanospheres with C-peptide or pentapeptide (C5) was carried out after preliminary activation of the carboxylic groups of glutamic acid. The nanospheres were prepared in 0.01 M Na-borate solution, pH 8.4, and then transferred into 0.01 M МES buffer, pH 5.6, via dialysis using tube membrane (MWCO 30,000). 1.0 mL of suspension with a concentration of 1.0 mg/mL was mixed with a two-fold excess of NHS and EDC required to activate 20% of Glu-units. The activation was carried out at 4 °C for 40 min. The activated nanospheres were washed with 0.01 M PBS (pH 7.4) via ultrafiltration to remove the activating agents and side-products of activation. Then, 200 μg of C-peptide or 50 μg of C5 in 0.01 M PBS were added to the suspension of activated nanospheres and left for 2 h at 22 °C. The excess of the peptide was removed via dialysis using MWCO 30,000 membrane against 0.01 M PBS. The amount of immobilized peptide was calculated as a difference between initial and unbound amounts of peptide. The quantity of unbound peptides was determined by means of HPLC analysis with UV-detection (λ = 210 nm). CIM DEAE disk of 3 mm × 12 mm i.d. (BIA Separations, Ajdovscina, Slovenia) was applied as stationary phase for this analysis. 0.01 M PBS, pH 7.0 (eluent A) and 0.01 M PBS, pH 7.0, containing 1 M NaCl (eluent B) were used as mobile phases. The flow rate was 0.5 mL/min. Injection volume was 20 µL. The HPLC analysis was carried out under gradient elution: 0–2 min—100% eluent A, 2–17 min—0–100% eluent B. Retention time for C-peptide was 9.8 min and for C5—0.9 min.

Immobilization efficiency (*IE*) was calculated as follows:

*IE* = (*m_i_ − m_s_*)/*m_i_* × 100% (1)

where *m_i_*—initial peptide amount (mg), *m_s_*—amount of unreacted peptide in solution (mg).

#### 2.2.6. Encapsulation of C-Peptide

The encapsulation of peptides was carried out via addition of 100 μL of peptide solution with chosen concentration (in a range 0.1–1.0 mg) into suspension of KF1-3 nanospheres immediately after their redispersion in 0.01 M PBS, pH 7.4. After that, the mixture was left at 4 °C for 20 h. Encapsulation of C5 was performed using 100 µL of peptide solution with concentration 1.0 mg/mL. Loading capacity (*LC*) and encapsulation efficiency (*EE*) were calculated using following equations:

*LC* = (*m_i_* − *m_s_*)/*m_NP_* (2)

*EE* = (*m_i_* − *m_s_*)/*m_i_* × 100% (3)

where *m_i_*—initial C-peptide amount (mg), *m_s_*—amount of non-encapsulated C-peptide in solution (mg), *m_NP_*—amount of nanospheres (mg).

The amount of encapsulated peptides was determined as difference between initial and non-encapsulated peptide amounts. The non-encapsulated peptides were removed from solution via ultrafiltration with the use of tubes supplied with membranes of MWCO 10,000 (used for C-peptide) and 3000 (used for C5). The filtrates were collected and lyophilized and then analyzed as described in Section 2.2.5.

In order to prepare the C-peptide loaded nanospheres with additional coverage with heparin, 1 mL of heparin in PBS with concentration of 1 mg/mL was added to 1 mL of preliminary prepared formulation in PBS with concentration of nanospheres equal to 2 mg/mL. Amount of encapsulated C-peptide in the experiments was varied from 100 to 600 µg.

#### 2.2.7. Drug Release

The release of C-peptide from nanospheres was studied at model physiological conditions, namely 0.01 M PBS, pH 7.4. 1.0 mL of dispersion of nanospheres (conc. 1.0 mg/mL) containing different loaded amount of C-peptide (from 150 to 600 µg/mg of nanospheres) was incubated at 37 °C. After predetermined time intervals, free C-peptide was separated from nanospheres by ultracentrifugation using microtube fitted with a membrane of 30,000 MWCO. The procedure was carried out 4 times with water and the filtered solution was freeze-dried and dissolved in 100 µL of water. The amount of released C-peptide was determined as described in Section 2.2.5.

To determine the stability of colloid system to aggregation as well as drug self-leakage, the suspension of loaded particles in 0.01 M PBS, pH 7.4, was incubated at 4 °C during three months. The supernatant was analyzed by ion-exchange HPLC (Section 2.2.5) to monitor the peptide self-release under storage conditions.

#### 2.2.8. Cell Culture Experiments

HEK-293 (human embryonic kidney) cell line were purchased from German Collection of Microorganisms and Cell Culture (DSMZ). Cells were cultivated in Dulbecco’s Modified Eagle Medium (DMEM, Sigma-Aldrich GmbH, Munich, Germany) supplemented with 10% (*v*/*v*) fetal calf serum (FCS, Biochrom GmbH, Berlin, Germany) and 1% (*v*/*v*) penicillin/streptomycin (P/S, Biochrom GmbH, Germany) in humidified environment at 37 °C/5% CO_2_. The medium was changed 3 times per week and the cells were subcultivated before reaching confluence using trypsin (Biochrom GmbH, Germany).

Eight × 10^3^ cells per well were seeded in a 96-well plate (100 μL/well) in DMEM culture medium and cultivated under a humidified atmosphere of 5% CO_2_ at 37 °C. After culturing for 24 h, the medium was replaced with the culture medium containing test nanospheres of different concentration (8–500 μg/mL). The viability was determined after 72 h treatment using CTB Assay (Promega, Mannheim, Germany). Culture medium was removed and 200 μL of CTB solution (10% stock solution in basal DMEM medium) were added to each well and incubated for 90 min. The number of viable cells was quantified by measuring fluorescence intensity (λ_ex_ = 544, λ_em_ = 590 nm) using a microplate reader (Fluoroscan Ascent, Thermo Fisher Scientific Inc., Waltham, MA, USA). Relative cell viability (%) was determined by comparing the fluorescence signals with control wells containing untreated cells. Data are presented as average ± SD (*n* = 4).

#### 2.2.9. Microcalorimetric Ex Vivo Assay of Na^+^/K^+^-ATPase on Living Erythrocytes

The study was approved by the local ethical committee of Saint-Petersburg State University and was performed in accordance with the relevant guidelines and ethical standards. The volunteers were 25–39 years (males, *n* = 5) and recruited by the laboratory staff of the Multidisciplinary Clinical Centre of Saint-Petersburg State University. Whole venous blood (10 mL) was collected from healthy volunteers after overnight fasting (12 h) by venepuncture directly into vacutainer tubes (Becton Dickinson, Plymouth, UK) containing heparin. After centrifugation (3000 rpm for 10 min), the plasma, buffy coat, and uppermost layer of erythrocytes were removed by aspiration. The remaining erythrocytes were resuspended and washed three times by inverting the tube to mix in a 10-excess physiological saline NaCl solution (0.9%) and centrifuged for 10 min at 2000 rpm. After the final wash, the hematocrit of the isolated erythrocytes was measured. Finally, living erythrocytes were resuspended in NaCl solution (0.9%) containing 5.5 mM glucose. Human C-peptide and C5 in different preparations were tested at physiological postprandial concentrations of 6 nmol/L.

Microcalorimetric experiments were performed using an isothermal titration calorimeter Nano ITC 2G set at 298.15 K (TA, New Castle, DE, USA). Each experiment consisted of single injection (5 μL) from a 100-μL rotating syringe of a solution containing different formulations of C-peptide or C-terminal pentapeptide (C5) into the microcalorimetric reaction cell (1 mL) charged with 500 μL of suspension of erythrocytes with concentration 1.1 × 10^9^ cells per mL. Quite low stirring rate (60 rpm) was necessary to avoid the damage of living erythrocytes. Heat production by the erythrocytes was recorded versus time. The steady state was reached after at least 1 h of incubation. Then, the tested compound was added to the suspension and changes in heat production were recorded. The heat of reaction was corrected for the heat of dilution of the guest solution, determined in the separate experiments. Ouabain (15 mmol/L) specific inhibitor of Na^+^/K^+^-ATPase was used to verify that heat production effects of C-peptide or C5 peptide and their formulations attributed to Na^+^/K^+^-ATPase activity. All solutions were degassed prior to titration experiment. Each injection generated a heat burst curve (μJ per second), the area under which was determined by integration using Nano ITC analyze software that gave the measure of the heat of reaction associated with the injection. Each sample was measured for three times.

All experiments were performed at 25 °C. The difference between heat production (µJ) in the steady state before and after the addition of respective test substances corresponds to the erythrocyte Na^+^/K^+^-ATPase activity normalized to sample volume (mL) and hematocrit (%). Sample volume, C-peptide amount and hematocrit remained constant for each measurement.

## 3. Results and Discussion

### 3.1. Polymerization and Polymer Characterization

**Figure 1:**
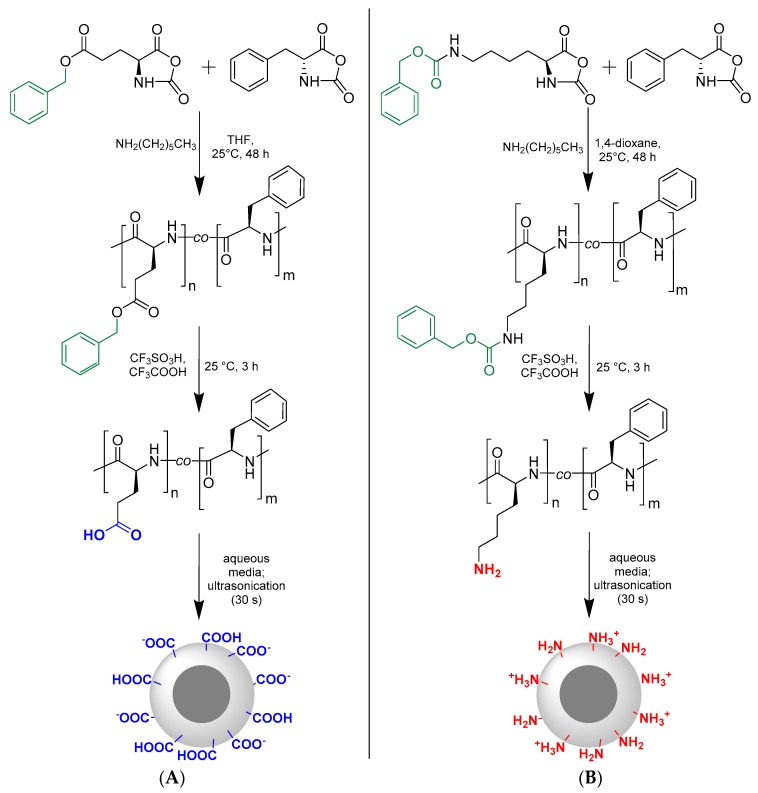
Scheme of polymerization and preparation of self-assembled nanospheres: (**A**) P(Glu-*co*-dPhe); (**B**) P(Lys-*co*-dPhe).

The synthesis of random copolymers P(Glu-*co*-dPhe) and P(Lys-*co*-dPhe) was carried out using ring-opening polymerization (ROP) of *N*-carboxyanhydrides of l-Glu(OBzl)/l-Lys(Z) and d-Phe (Figure 1). The series of random copolymers were synthesized with different monomer ratios (Table 1). The molecular weight (*M_w_* and *M_n_*) and dispersity (*Ð*) of protected copolymers was determined using size-exclusion chromatography (SEC) (Table 1). All synthesized polymers were characterized with low dispersity (1.07 ≤ *Ð* ≤ 1.29). *Ð* values were minimal when the ratio of l-Glu(OBzl)/l-Lys(Z) NCA to d-Phe NCA was 1/1 and maximal for the ratio 8/1.

The preparation of amphiphilic copolymers was achieved after the removal of *Z*- and *Bzl*-protective groups. After deprotection the polymers became to be insoluble in water and demonstrated the tendency to self-assembly. The deprotection was proved by ^1^H NMR spectroscopy (Figure S2 of Supplementary Materials).

The composition of synthesized polymers was proved by quantitative HPLC analysis of the samples prepared via total acidic hydrolysis of polypeptides up to free amino acids. The results obtained are presented in Table 2. Additionally, for copolymers based on glutamic acid and phenylalanine the composition was calculated from ^1^H NMR spectra (Table 2, Figure S2 of Supplementary Materials). Polymer composition established by two methods was in a good agreement. For sample EF1, prepared with initial [Lys(Z) NCA]/[Phe NCA] ratios equal to 1/1, the experimentally found composition was close to the theoretical one. An increase of the ratio of glutamic acid monomer in polymerization mixture was followed by partial diminishing of Glu units in final copolymers. In turn, the polymer compositions for KF1 and KF2 samples were matched well with that of monomer mixture. An increase of [Lys(Z) NCA]/[Phe NCA] to 8/1 was followed by the partial enrichment of final polypeptide with lysine. For copolymers based on lysine and phenylalanine the determination of polymer composition by ^1^H NMR was impossible because of broadening and overlapping of reference signals.

### 3.2. Preparation and Characterization of Nanospheres

There are many different techniques for the preparation of polymer nanoparticles such as solvent evaporation, nanoprecipitation, emulsification/solvent diffusion (ESD), high-pressure homogenization, phase inversion (dialysis), film rehydration, etc. [31]. The approach selection straightly depends on the polymer properties (chemical nature, charge, hydrophilicity/hydrophobicity, swelling, reactivation, pH-dependency, etc.). In our case, the phase inversion (dialysis) method was chosen because of its simplicity and suitability to prepare self-assembled nanosystems [32,33,34]. The hydrodynamic diameter of nanospheres obtained was determined using dynamic light scattering (DLS) method at different pH.

**Figure 2:**
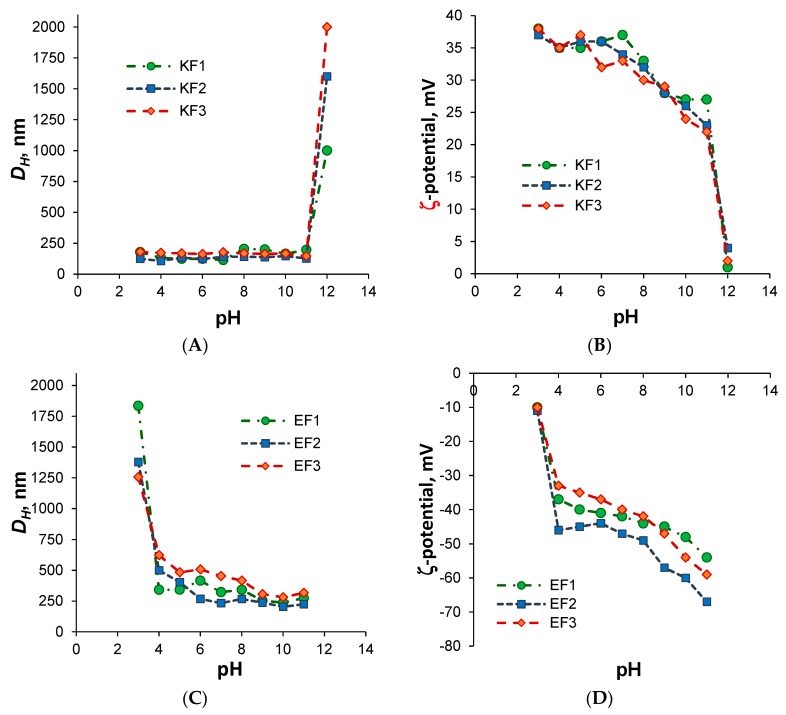
Dependence of hydrodynamic diameter and ζ-potential of polypeptide nanospheres on pH: (**A**,**B**)—P(Lys-*co*-dPhe); (**C**,**D**)—P(Glu-*co*-dPhe).

The hydrodynamic diameter of P(Lys-*co*-dPhe) nanospheres was varied from 110 to 220 nm for the different polymer composition in the pH range from 3 to 11 (Figure 2A). The increase of pH up to 12 induced the immediate aggregation of nanospheres that was a result of the loss of particle surface charge, which was necessary to stabilize the colloid system towards the aggregation (Figure 2B).

The similar tendency was observed for P(Glu-*co*-dPhe) nanospheres but, in this case, the isoelectric point was at pH 3 (Figure 2D). In the range of pH 5–11 the nanospheres were stable. Usually, the colloid system is counted as stable if ζ-potential absolute value is higher than 30 mV [35]. In our case, both kinds of nanospheres met this requirement. For P(Glu-*co*-dPhe), the nanospheres with the smallest hydrodynamic diameter (350–210 nm depending on the polymer composition) were formed in the range of pH from 7 to 11 (Figure 2C). This fact can be explained by better ionization of carboxylic groups at pH higher than 7. The better ionization is followed by the repulsion of like-charged polymer chains that in turn favored to the self-assembly into the smaller-sized nanospheres.

Additionally, the dependence of average hydrodynamic diameter of nanospheres on the sample dilution was evaluated. The dilution of dispersion with starting concentration of 2 mg/mL in 250 times (up to 0.008 mg/mL) did not follow with the change in *D_H_* of nanoparticles. All colloid systems were stable under incubation in 0.01 M PBS, pH 7.4, during three months without changing their hydrodynamic diameter, aggregation and sedimentation.

**Figure 3:**
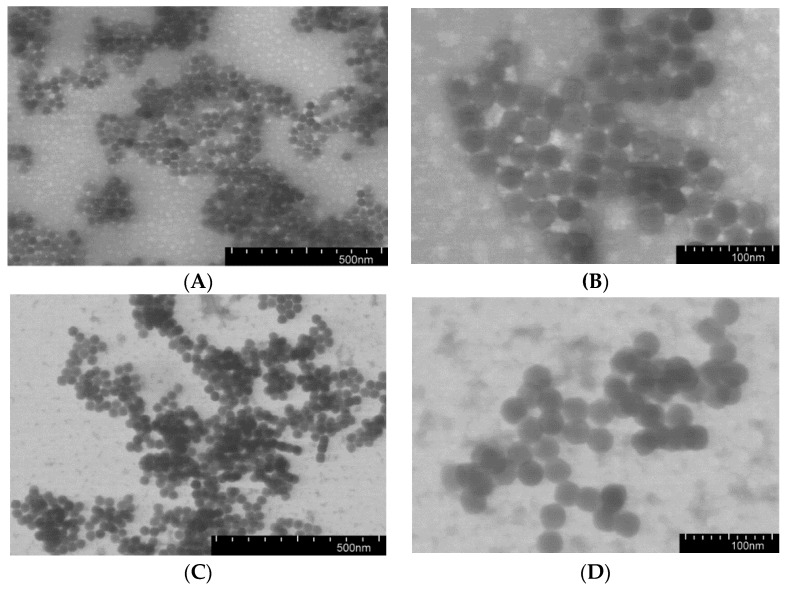
Transmission electron microscopy (TEM) images of P(Glu-*co*-dPhe) (**A**,**B**) and P(Lys-*co*-dPhe) (**C**,**D**) nanospheres (samples EF2 and KF2).

The nanospheres prepared in 0.01 M PBS buffer, pH 7.4, were investigated by transmission electron microscopy (TEM) (Figure 3). The size of polymer nanoparticles observed by TEM in a dry state was about 30 ± 5 nm. Such difference between hydrodynamic diameter registered in aqueous media by DLS and diameter of nanospheres determined by TEM in a dry state is known for soft self-assembled materials [36,37]. This effect is related to the volatilization of water during the TEM grid drying before analysis. In aqueous media, the hydrodynamic diameter of nanospheres is provided by the expansion of charged water-soluble-like charged fragments (Lys or Glu) because of their repulsion.

### 3.3. In Vitro Biodegradation Study

**Figure 4:**
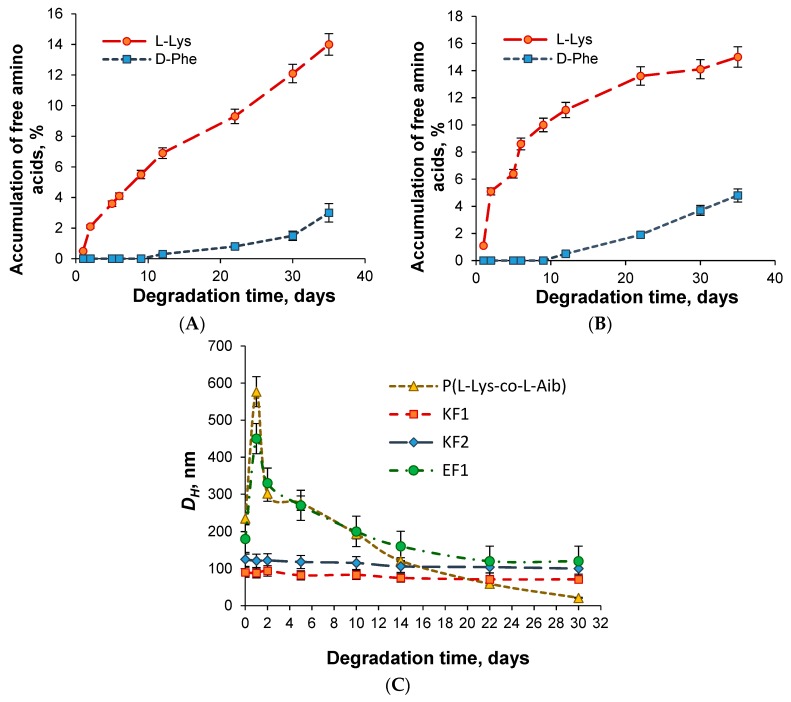
Degradation of P(Lys-*co*-dPhe) nanospheres on time: (**A**,**B**) accumulation of free amino acids during the process for samples KF1 and KF2, respectively (HPLC); (**C**) Decrease of hydrodynamic size on time (DLS). Conditions of biodegradation*:* incubation was performed in 0.01 M PBS, pH 7.4, and at 37 °C; concentration of nanospheres was 1.0 mg/mL; concentration of papain was 0.5 mg/mL.

P(Lys-*co*-dPhe) nanospheres were chosen to evaluate the biodegradation process in vitro. The process was performed in 0.01 M PBS, pH 7.4, containing papain as model enzyme. Papain, being a thiol proteinase of plant origin, represents the analogue of lysosomal endopeptidase cathepsin B regarding to its activity towards the bonds formed between different α-amino acids. The biodegradation process was carried out in two ways: (1) as a function of free amino acids accumulation in the reaction mixture during the time and (2) as a function of change in hydrodynamic diameter of particles (Figure 4). According to HPLC data only 14% of l-Lys and 3% of d-Phe were detected for sample KF1 after a month of particle incubation with papain at 37 °C. For sample KF2, 15% of l-Lys and 5% of d-Phe were determined for the same time.

The monitoring of hydrodynamic diameter of nanospheres during a month allowed for detection of decrease in this parameter. In particular, 15% and 17% decrease in *D_H_* was established for KF1 and KF2 samples and 34% for EF1 one. The results obtained were compared to the data on biodegradation of random nanospheres based on copolymer of l-lysine and l-aminoisobutyric acid (P(Lys-*co*-Aib)), containing 29 mol% Aib and developed recently in our group [37]. Aib represents natural but non-coded amino acid, which sometimes is also used to improve the ability to enzymatic degradation [38]. As it can be seen from Figure 4B, the hydrodynamic diameters were drastically diminished of the P(Lys-*co*-Aib)-based nanospheres incubated at the same conditions and for the same time period. The total decrease in *D_H_* corresponded to 87%. Thus, the developed P(Glu-*co*-dPhe) and P(Lys-*co*-dPhe) nanospheres can be counted as biomaterials with increased stability to biodegradation.

### 3.4. Surface Modification

**Figure 5:**
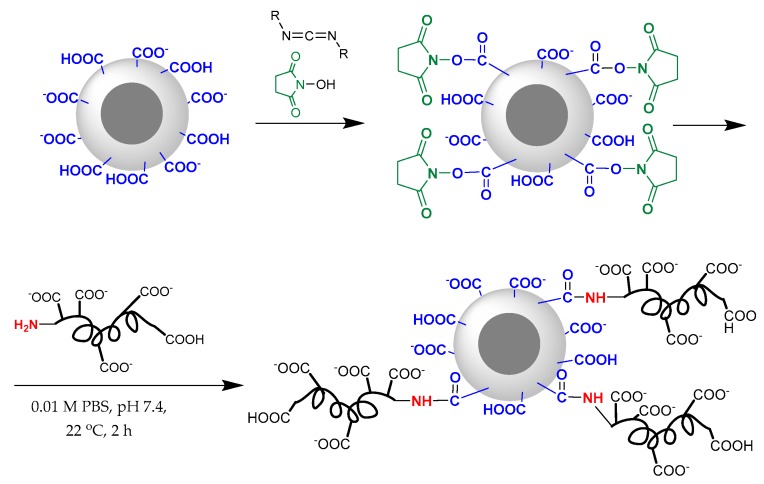
Scheme of covalent modification of P(Glu-*co*-dPhe) particle surface.

The presence of chemically reactive functional groups on the surface of poly(amino acid) nanospheres allowed the modification of their surface by different biomolecules including peptides. In present work, for preparation of C-peptide formulation with preserved peptide biological activity and prolonged stability we chose the method of one-point attachment of C-peptide and its short C5 fragment (EGSLQ) via the reaction of α-amino group of N-terminal amino acid with carboxylic group of P(Glu-*co*-dPhe) nanospheres. This approach includes an activation of carboxylic groups with water soluble carbodiimide and *N*-hydroxysuccinimide (NHS) to form activated NHS ester (Figure 5).

The samples EF1, EF2 and EF3 differ in the content of hydrophilic and hydrophobic units were modified with C-peptide (Table 3). For all nanospheres the reaction of covalent immobilization was carried out under the same conditions (2 h at 22 °C) and amount of activated carboxylic groups (20 mol%). It was established that the quantity of the bound C-peptide depended on polymer composition. The highest amount of immobilized C-peptide was determined for the sample EF2 containing intermediate ratio of Glu and Phe units. In this case, 48 µg of C-peptide per mg of nanospheres was attached onto the nanoparticle’s surface.

The low immobilization efficiency of C-peptide on the surface of EF1 nanospheres may be related to the lowest amount of Glu units among the tested polymers. In turn, very high content of Glu units in the polymer probably favored to the higher repulsion of highly negative charged nanospheres and like-charged C-peptide.

EF2 nanospheres were selected for the immobilization of C5. It is known that the functional groups of small molecules are characterized with better reactive accessibility comparatively to the more elongated ones. Taking this into account, the initial C5 amount used for immobilization was reduced twice comparatively to C-peptide (from 200 to 100 µg). Despite the fact that immobilization efficiency was only 16%, the molar amount of immobilized C5 was appeared to be higher than this established for C-peptide.

The developed delivery systems were tested for the storage stability. The samples with concentration of 1 mg/mL were stored at 4 °C during three months. The monitoring of hydrodynamic diameter as well as free C-peptide in 0.01 M PBS, pH 7.4, revealed the high stability of the formulation at low temperatures. In particular, *D_H_* change or aggregation of nanospheres, as well as drug leakage were not detected.

### 3.5. C-Peptide Encapsulation

**Figure 6:**
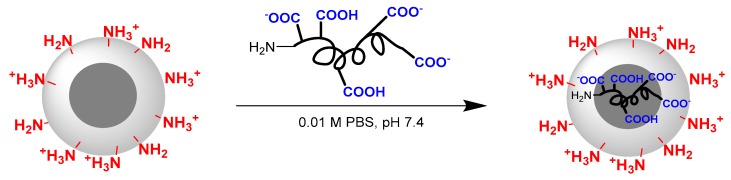
Scheme of encapsulation of C-peptide into the P(Lys-*co*-dPhe) nanospheres.

Encapsulation of C-peptide into P(Lys-*co*-dPhe) nanospheres represents an alternative approach for the preparation of a long-acting peptide formulation. P(Lys-*co*-dPhe) nanospheres were chosen because of their positive charge, which can provide the ionic interactions with highly negative charged C-peptide (Figure 6).

For the first step, the encapsulation efficiency (EE) and loading content (LC) on the polymer composition were analyzed at constant initial amount of C-peptide (100 μg). All tested polymer nanospheres demonstrated very high loading efficiency (Table 4). The lowest loading efficiency (89.5%) was observed for the nanospheres based on KF1 sample containing the lowest amount of lysine units in the polymer chains. For KF2 and KF3 samples, containing higher content of lysine than KF1, the values of *EE* were very close to 95%. The encapsulation efficiency of C5 into KF3 nanospheres was similar to that established for C-peptide.

Additionally, the hydrodynamic diameter of nanospheres loaded with C-peptide was measured in 0.01 M PBS, pH 7.4, and compared (Table 4). It was found that the *D_H_* values were increased with lysine enrichment in polymer chains. The smallest nanospheres were formed from KF1 polypeptide whereas the largest ones were obtained for KF3 sample. The higher content of hydrophobic monomer favors to compaction of the particles during the polymer self-assembling. In turn, the high number of charged fragments tends to repulse each other and, as a result, the hydrodynamic diameter of self-assembled particles is increased.

**Figure 7:**
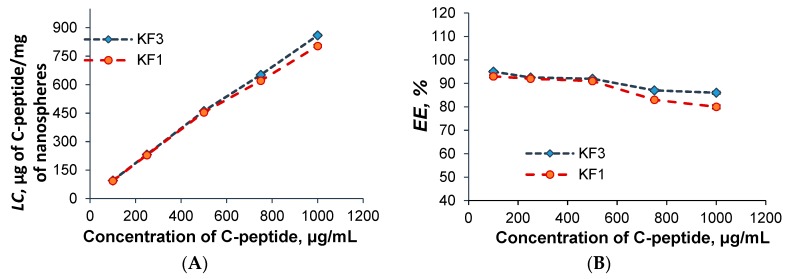
Dependence of loading capacity (LC) (**A**) and encapsulation efficiency (EE) (**B**) on initial concentration of C-peptide.

For the study of the dependences of *LC* and *EE* on initial C-peptide concentration, two samples containing the highest and the lowest amount of hydrophobic amino acid, e.g., KF1 and KF3, were selected. It was established that an increase of initial concentration of C-peptide from 100 µg/mL to 1000 µg/mL followed by nearly linear increase of loading capacity (Figure 7A). The maximum *LC* of C-peptide for KF1 and KF3 nanospheres were equal to 804 ± 6 and 860 ± 5 μg of C-peptide/mg of nanoparticles, respectively. When the initial C-peptide concentration was higher than 1000 µg/mL the aggregation of particles was detected. This fact can be attributed to the partial neutralization of lysine positive charge of the P(Lys-*co*-dPhe) particles with negative charge of C-peptide. It was proved by an intense decrease of ζ-potential from +40 to +20 mV.

In turn, the encapsulation efficiency for KF1 and KF3 particles was just slightly reduced from 89.5% and 95.0%, determined at initial drug concentration of 100 µg/mL, to 80.2% and 86.4% detected for concentration 1000 µg/mL, respectively (Figure 7B).

**Figure 8:**
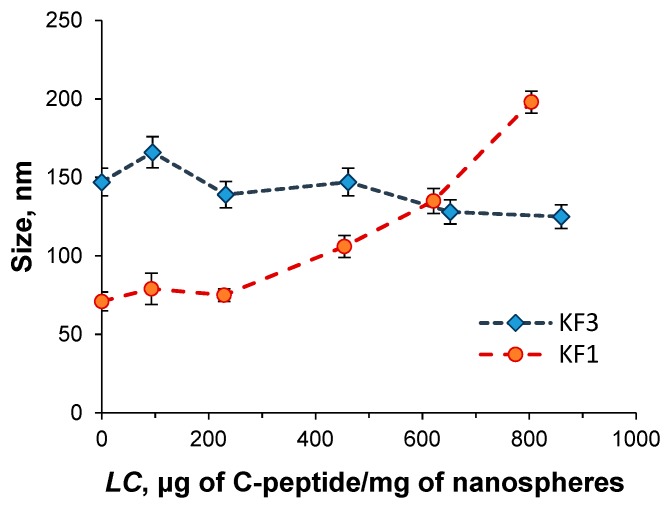
Dependence of nanosphere’s hydrodynamic diameter on C-peptide loading content.

Since the high drug loading may affect the particle size, the hydrodynamic diameter of KF1 and KF3 nanospheres loaded with the different quantity of C-peptide were analyzed (Figure 8). For nanospheres with the maximal lysine content (KF3) the hydrodynamic diameter did not depend on C-peptide loading amount. On the contrary, the hydrodynamic diameter of KF1 nanospheres, containing the minimal lysine amount, was increased with the growth of drug loading. At maximal value of *LC* equal to 804 μg of C-peptide per mg of polymer nanospheres, ζ-potential of KF1 particles decreased to +30 mV that can be attributed to the partial charge neutralization because of C-peptide loading comparatively to the KF3 nanospheres enriched with lysine units.

As for C-peptide covalently bound to the nanospheres, the encapsulated forms of C-peptide demonstrated also high storage stability towards the preservation of colloid’s properties at low temperature (4 °C). During three months the changes in hydrodynamic diameter or precipitation of formulation were not observed. Moreover, the monitoring of free C-peptide in solution revealed the leakage of 8 ± 1% of the drug from KF3 nanospheres with high loading of C-peptide (604 µg/mg). At the same conditions, C-peptide leakage at 37 °C was found to be 30 ± 1%. For KF3 nanospheres with high loading of C-peptide covered with heparin the drug leakage at low temperature was not detected.

### 3.6. Drug Release

**Figure 9:**
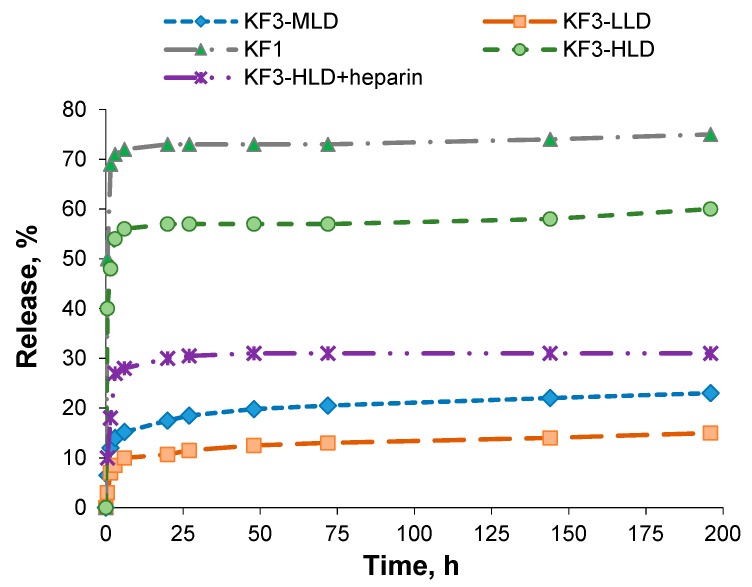
Kinetics of C-peptide release from P(Lys-*co*-dPhe) nanospheres (37 °C, 0.01 M PBS, pH 7.4).

Along with loading content and encapsulation efficiency the drug release kinetics is also one of the important properties of drug delivery systems. For the systems under study, the drug release was studied in 0.01 М PBS, pH 7.4, at 37 °C for KF1 and KF3 nanospheres. The last kind of nanospheres was applied as three formulations with different loaded amount of C-peptide. The summary on tested formulations is presented in Table 5. As it can be seen from Figure 9, during 6 h 57 and 73% of C-peptide were released from KF3 and KF1 nanospheres, respectively. After this time, no further C-peptide release was detected in model conditions. The increase of lysine monomer content in the polymer provided higher retention of peptide inside the nanospheres. Thus, KF3 nanospheres as delivery systems seems to be more preferable.

KF3 formulations with lower C-peptide loading demonstrated similar drug release profile (Figure 9) however the amount of released peptide were less than for nanospheres with high drug loading (Table 5).

To diminish the release ratio, the nanospheres with high C-peptide loading (KF3-HLD) were additionally covered with heparin. The covering was evidenced by an inversion of their average ζ-potential value from positive (+35) to negative (−38 mV). The covering of KF3-HLD was followed by substitution of ~20% of surface adsorbed C-peptide with heparin. As it was expected, the heparin envelope prevented the intensive drug leakage and the maximal release ratio did not exceed 31%.

The release observed in model buffer medium can be explained by the leakage of C-peptide, weakly retained due to the ionic interactions, from close to the nanosphere’s surface and directly from the surface. The comparable amounts of C-peptide retained inside KF3-HLD and KF3-MLD systems after two weeks of incubation in PBS (Table 5) give reasons for suggestion that retained amounts are deeply buried inside the polymer nanospheres. The release of this portion of C-peptide is possible only as response on some stimuli, for example, due to partial polymer envelope degradation or the nanosphere’s disassembly because of interaction with polyanions, proteins, some other peptides, etc. Since the used for the release study buffer medium could not supply any extra stimuli, the further release was suspended. Contrary to the applied model buffer medium the biological fluids can provide many events inducing the second phase of C-peptide release.

However, as it is seen from data presented in Table 5, the highest amount of retained C-peptide (335 µg/mg of nanospheres) was detected for KF3-HLD + heparin formulation. The covering with heparin allows for the safety not only deeply buried C-peptide but also drug distributed near to the surface. Therefore, the covering of loaded nanospheres with heparin favored to C-peptide preservation in the polymer nanospheres. Overall, the covering of polypeptide nanospheres with polysaccharide provides the additional barrier for proteases and can additionally prolong the stability of drug formulation.

Thus, among the developed and tested formulations at least three candidates can be marked out as most potential encapsulated C-peptide delivery systems: (1) KF3-HLD which provides high release rate at the first step and has enough reserve for the second step; (2) KF3-MLD which supplies moderate release ratio at the first step and has enough reserve for the second one; (3) KF3-HLD + heparin which provides moderate release ratio at the first step and has high potential for the second step of release.

### 3.7. Cell Culture Experiments

**Figure 10:**
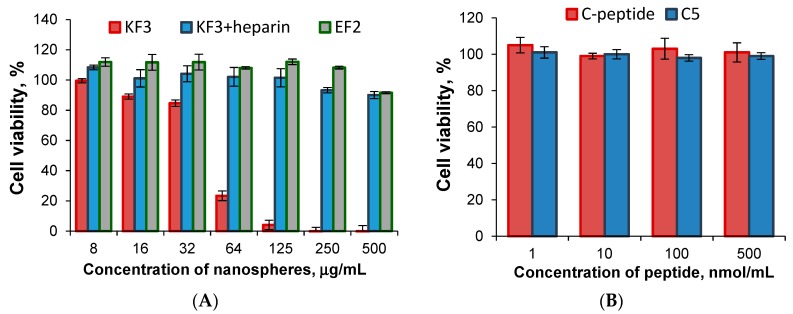
Cell viability (HEK-293) after their incubation for 3 days in presence of different nanospheres (**A**); C-peptide and C5 peptide (**B**).

The cytotoxicity of prepared nanospheres as well as C-peptide and short C5 peptide was tested with the use of human embryonic kidney cells (HEK-293) (Figure 10). In order to evaluate cell viability, the samples with the best characteristics and properties established in previous experiments were selected. These were KF3, KF3 covered with heparin and EF2 samples. The experiments were carried out during 3 days at concentration of nanospheres from 8 to 500 μg/mL. It was established that KF3 sample was not toxic up to the concentration equal to 32 μg/mL (viability 86%) and then the considerable cell death was detected. The same nanospheres but covered with heparin, as well as EF2 one, demonstrated no cytotoxicity at all tested concentrations (viability ≥ 90%).

**Figure 11:**
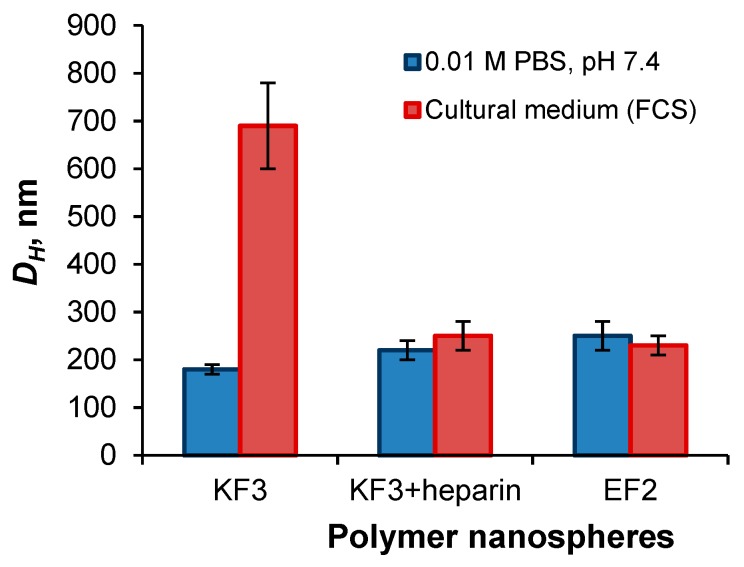
Stability of different nanospheres in culture medium.

The study of nanospheres stability in the DMEM+FCS culture medium within three days revealed an increase of particle hydrodynamic diameter (DLS) for KF3 sample (Figure 11). In turn, the negatively charged particles, namely KF3 covered with heparin and EF2, demonstrated stability in protein-containing culture medium. Thus, the cytotoxicity of KF3 may be caused by their high level of aggregation in culture medium [39].

### 3.8. Microcalorimetric Study

Na^+^/K^+^-adenosine triphosphatase (Na^+^/K^+^-ATPase) activity is low in various tissues and erythrocytes of Type I diabetic patients [40,41]. This enzyme activity plays a key role in the pathogenesis of diabetic neuropathy and the red cell deformability. Na^+^/K^+^-ATPase is a ubiquitously expressed transmembrane enzyme that sustains cellular homeostasis by keeping low Na^+^ and high K^+^ concentrations in cytosol. Cellular homeostasis is pivotal for maintenance of the membrane potential of all types of cells and acts by driving an active Na^+^ and K^+^ transport through the cellular membrane [42]. It is known that C-peptide improves the human vascular blood flow through a mechanism involving endothelial NO production and erythrocyte Na^+^/K^+^-ATPase activation [41]. Ohtomo et al. established that C-peptide stimulates Na^+^, K^+^-ATPase of renal tubule segments due to the activation of a receptor coupled to a pertussis toxin-sensitive G-protein with subsequent activation of Ca^2+^-dependent intracellular signaling pathways [43,44].

In our case, the biological effects of C-peptide and C5 formulations were studied using isothermal titration microcalorimetry method. The heat released via the Na^+^/K^+^-adenosine triphosphatase during ATP hydrolysis represents a signal of steady-state enzymatic activity. For this purpose, living erythrocytes were incubated with and without different formulations of C-peptide and C5 (ex vivo study). It was previously established that a concentration-dependent stimulation is observed in the range 1–10 nM of C-peptide [40]. In our case, C-peptide in different preparations were tested at physiological postprandial concentrations of 6 nM. To compare, C5 concentration was chosen to be equal to concentration of C-peptide.

The results of calorimetric titration of erythrocyte suspension by tested formulations of C-peptide are shown in Table 6. Ouabain is specific inhibitor of Na^+^/K^+^-ATPase, so it was used to prove that observed heat production is attributed to Na^+^/K^+^-ATPase activity in presence of C-peptide. As it was expected, no heat release was observed under incubation of erythrocytes with C-peptide in presence of ouabain. Both C-peptide and its C-terminal fragment C5 stimulated Na^+^/K^+^ ATPase in erythrocytes but in the case of C5 activation effect was slightly higher. The high activity of C-terminal pentapeptide fragment of C-peptide was proved earlier both ex vivo and in vivo. Using rat C-peptide Ohtomo et al. has shown that Na^+^/K^+^-ATPase activity of C-terminal pentapeptide (EVARQ) was found to 103% of the intact molecule’s activity [43]. Nordquist et al. showed that administration of the rat C-peptide fragment EVARQ has similar effects on glomerular filtration rate and blood glucose levels as the intact C-peptide molecule in rats [26].

In our case, the biological effect of tested C-peptide formulations was different. For encapsulated systems the drug loading, and consequently the release rate, influenced on measured heat release. The highest value of ∆*H* was detected when C-peptide loading was high (604 µg/mg of nanospheres). The observed high effect can be a sum of both C-peptide burst release and Na^+^/K^+^-ATPase activation by the released C-peptide. In turn, the application of low-loaded nanospheres (147 ± 4 µg/mg of nanospheres) that demonstrated a very slow release of C-peptide, gave much lower heat production. This result can be attributed to a much less amount of free C-peptide involved in Na^+^/K^+^-ATPase activation. A comparable result was also observed for nanospheres with a low loading of C5 (143 ± 3 µg/mg of nanospheres).

The covalent immobilization of C-peptide and C5 on the particle surface was carried out in the same way to provide the one-point attachment of peptide through its N-terminus. Firstly, such immobilization ensures the peptide sequence remains untouched and secondly, the accessibility to C-terminus, which is important for biological activity. The formed amide bond between peptide and polymer side-chain is stable in the peptidase-free media and, therefore, no release is possible in PBS. Thus, the heat release measured for immobilized forms of peptides is responsible for Na^+^/K^+^-ATPase activation with C-peptide/C5 located at surface of nanospheres. As it can be seen from data presented in Table 6, the activity of immobilized C-peptide was much higher than this determined for bound C5. The poor activity of C5 is conditioned by steric hindrances in interaction of short peptide located at the particle’s surface with erythrocytes. Contrary to C5, the accessibility of more elongated and better in solution exposed peptide is much higher. Thus, for this kind of formulation, the application of C-peptide-bearing nanospheres rather than that of C5 appears to be a worthy prospect.

## 4. Conclusions

In this work, the random amphiphilic polypeptides with prolonged stability to biodegradation were synthesized and characterized. For both anionic and cationic polymers, the pH of self-assembly did not influence the hydrodynamic size of nanospheres in a wide range of pH. All self-assembled nanospheres were stable in 0.01 M PBS at 4 °C (storage stability) at least three months. In culture medium-containing proteins, the anionic nanospheres were stable but the cationic ones were aggregated. This instability in the culture medium seems to be one of the main reasons of the cytotoxicity of the P(l-Lys-*co*-d-Phe) nanospheres. In turn, the stable in culture medium anionic particles did not demonstrate any cytotoxicity up to the concentration of 500 µg/mL. The successful application of the developed nanospheres for C-peptide and its short fragment C5 encapsulation and immobilization was demonstrated. Both formulations were stable under storage conditions for three months in regards of their size and C-peptide content. The developed novel formulations of C-peptide demonstrated high biological effects by stimulating Na^+^/K^+^-ATPase activity in erythrocytes and some can be considered for further examination in experimental animals.

## Figures and Tables

**Table 1:** Monomer ratios, polymer yields and characteristics of synthesized protected copolymers.

Sample	Initial Ratio of NKAs: [Glu(OBzl)/Lys(Z)]/[d-Phe]	Polymer Characteristics (SEC)			Polymer Yield, %
		*M_n_*	*M_w_*	*Ð*	
	P(Glu(OBzl)*_n_*-*co*-dPhe*_m_*)				
E(Bzl)F1	1/1	5600	6400	1.15	49
E(Bzl)F2	4/1	6700	8100	1.20	70
E(Bzl)F3	8/1	7100	9200	1.29	72
	P(Lys(Z)*_n_*-*co*-dPhe*_m_*)				
K(Z)F1	1/1	14,000	15,800	1.07	68
K(Z)F2	4/1	21,500	24,300	1.13	55
K(Z)F3	8/1	24,300	28,000	1.15	71

**Table 2:** Composition of amphiphilic random polypeptides and polymer yields after deprotection.

Sample	Determined Polymer Composition					
	HPLC			^1^H NMR		
	*n*	*m*	[Glu/Lys]/[Phe] Ratio	*n*	*m*	[Glu]/[Phe] Ratio
P(Glu*_n_*-*co*-dPhe*_m_*)						
EF1	17	14	1.2	16	15	1.1
EF2	33	11	3.0	37	10	3.7
EF3	38	7	5.4	45	8	5.6
P(Lys*_n_*-*co*-dPhe*_m_*)						
KF1	34	34	1.0	-	-	-
KF2	72	17	4.3	-	-	-
KF3	87	9	9.5	-	-	-

**Table 3:** Characteristics of nanospheres under C-peptide and C5 covalent immobilization. Conditions of immobilization*:* 0.01 M PBS, pH 7.4; 22 °С; 2 h.

Sample	Amount of Bound Peptide, μg/mg of Nanospheres	Amount of Bound Peptide, nmol/mg of Nanospheres	Immobilization Efficiency, %
C-peptide			
EF1	20 ± 4	5.5	10 ± 1
EF2	48 ± 5	13.3	24 ± 2
EF3	25 ± 3	6.9	13 ± 2
C5			
EF2	16 ± 2	30.0	16 ± 2

**Table 4:** Dependence of encapsulation efficiency, loading content and hydrodynamic diameter of nanospheres on polymer composition and peptide.

Sample	*D_Ho_* *, nm	*EE* **, %	*LC*, µg/mg of Particles	*D_H encaps_* *, nm	*PDI _encaps_* *
C-peptide					
KF1	71 ± 3	89.5 ± 0.6	89.5 ± 0.5	79 ± 8	0.24
KF2	96 ± 3	94.6 ± 1.0	94.6 ± 0.9	130 ± 20	0.16
KF3	150 ± 10	95.0 ± 1.1	95.0 ± 1.0	190 ± 20	0.14
C5					
KF3	150 ± 10	96.2 ± 0.7	96.2 ± 0.7	178 ± 15	0.13

* measured in 0.01 M PBS, pH 7.4; ** the initial amount of peptide taken for loading was 100 µg.

**Table 5:** Formulations applied for C-peptide release study.

Sample	Amount of C-Peptide Encapsulated, µg/mg of Nanospheres	Amount of C-Peptide Retained after 14 Days Release, µg/mg of Nanospheres
KF1	581 ± 3	157 ± 4
KF3-HLD	604 ± 4	259 ± 5
KF3-MLD	285 ± 5	225 ± 7
KF3-LLD	147 ± 4	128 ± 3
KF3-HLD + heparin	483 ± 8	335 ± 10

*Abbreviations:* HLD—high loaded, MLD—middle loaded and LLD—low loaded.

**Table 6:** Results of isothermal titration microcalorimetry experiments.

#	Experimental Condition	∆*H*, μJ Normalized to Control
1	C-peptide + ouabain	0 *
2	C-peptide	−102 ± 7 *
3	C5	−136 ± 9 *
4	C-peptide encapsulated in KF3 nanospheres (HLD)	−265 ± 19 **
5	C-peptide encapsulated in KF3 nanospheres (LLD)	−65 ± 5 **
6	C-peptide immobilized on the surface of EF2 nanospheres	−213 ± 16 ***
7	C5 encapsulated in KF3 nanospheres (LLD)	−54 ± 6 **
8	C5 immobilized on the surface of EF2 nanospheres	−15 ± 4 ***

* 0.9% NaCl was used as a control; ** KF3 nanoparticles in 0.9% NaCl was used as a control; *** EF2 nanoparticles in 0.9% NaCl was used as a control.

## Supplementary Materials

The following are available online at http://www.mdpi.com/1999-4923/11/1/27/s1, Figure S1. Scheme of synthesis of NCAs of γ-Bzl-l-Glu (A), ε-Z-l-Lys (B) and d-Phe (C), Figure S2. 1H NMR spectra of P(Glu(OBzl)-*co*-Phe) (A) and P(Glu-*co*-Phe) (B) obtained after deprotection of γ-glutamic carboxyls.
