# Supplementary material for: Novel Formulations of C-Peptide with Long-Acting Therapeutic Potential for Treatment of Diabetic Complications
Source: Pharmaceutics. 2019 Jan 11;11(1):27. doi: 10.3390/pharmaceutics11010027 (PMC6359607; doi:10.3390/pharmaceutics11010027)
Supplement: Supplementary file 1 [file pharmaceutics-11-00027-s001.pdf]

pharmaceutics

MDPI

Supplementary Materials: Novel Formulations of C-Peptide with Long-Acting Therapeutic Potential for Treatment of Diabetic Complications

Natalia Zashikhina, Vladimir Sharoyko, Mariia Antipchik, Irina Tarasenko, Yurii Anufrikov, Antonina Lavrentieva, Tatiana Tennikova and Evgenia Korzhikova-Vlakh

(A)

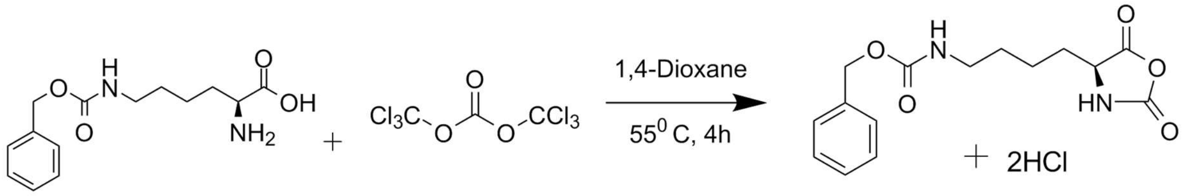

(B)

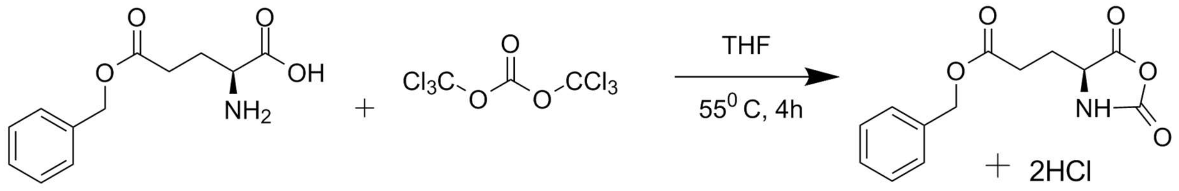

(C)

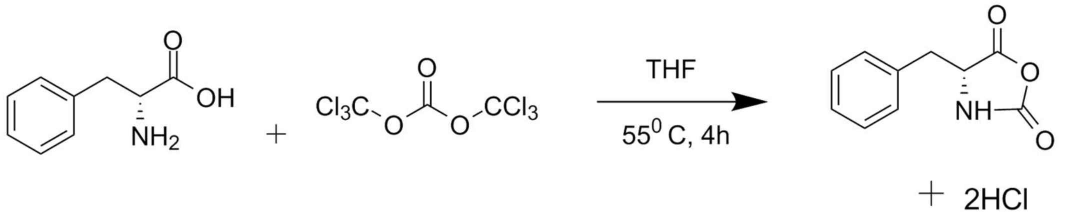

Figure S1. Scheme of synthesis of NCAs of γ-Bzl-L-Glu *(A)*, ε-Z-L-Lys *(B)* and D-Phe *(C)*.

(A)

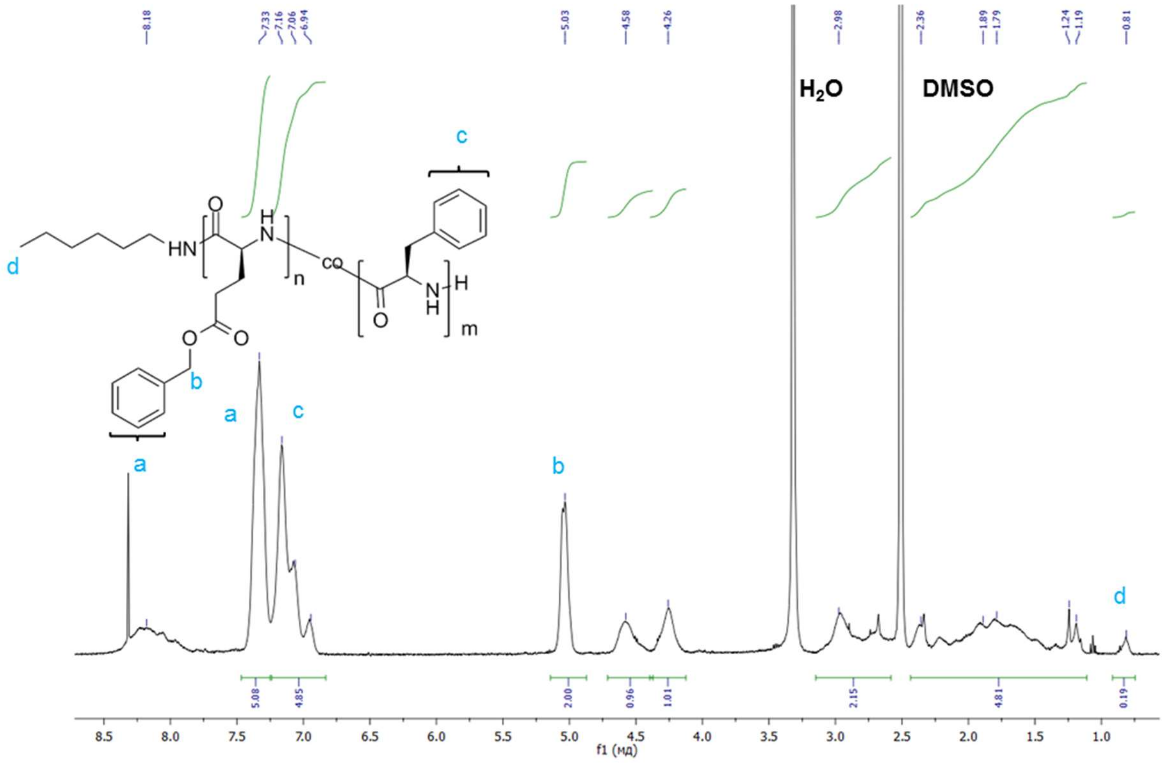

(B)

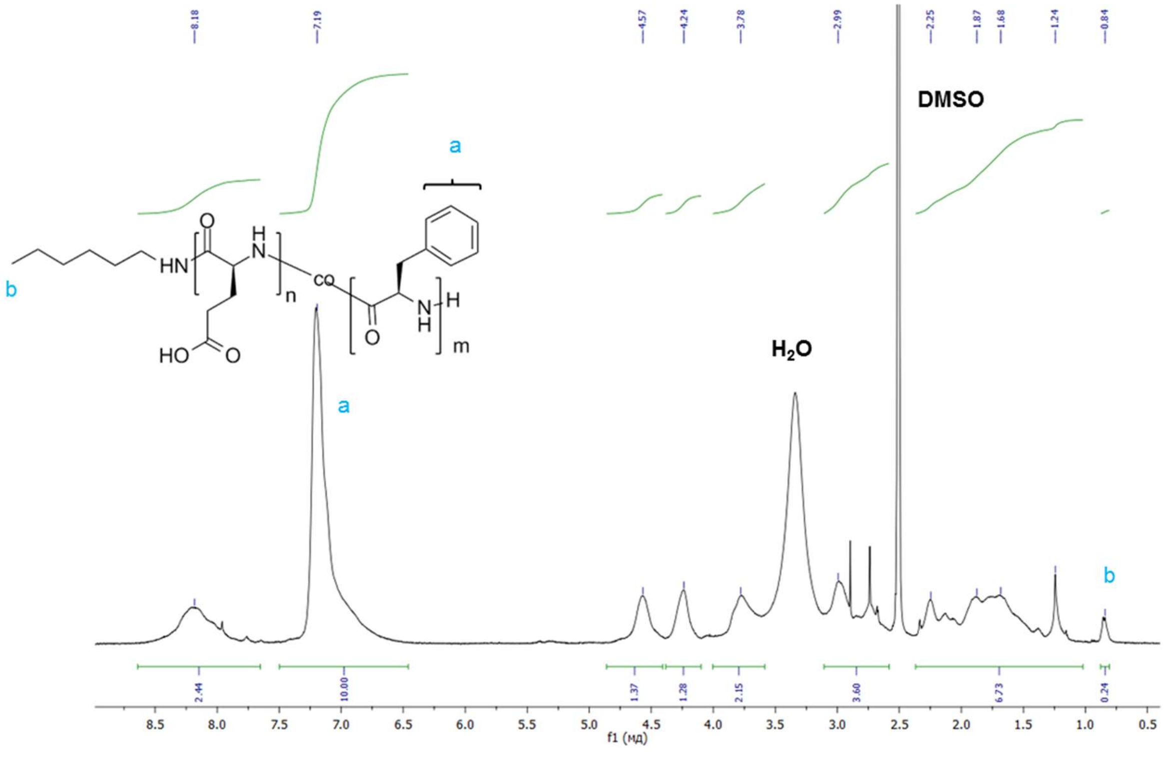

Figure S2. ^{1}H NMR spectra of P(Glu(OBzl)-*co*-Phe) *(A)* and P(Glu-*co*-Phe) *(B)* obtained after deprotection of γ-glutamic carboxyls.
